# Local Treatment of Cancer of the Mouth

**Published:** 1875-05

**Authors:** J. E. Nichols


### ARTICLE XII.

### *Local Treatment of Cancer of the Mouth.*

Dr. J. E. Nichols recommends, in the Chicago *Medical Journal,* the more frequent use of local applications in cancer. Of the cases he gives, the following two show his treatment:—

Case 1. Mrs. J. B., aged fifty years, sent for me December 10th, 1873. Has been scrofulous and an invalid several years. No cancer in her family. For several months a small, hard, painful tumor has been coming on the gums of incisor teeth and roof of the mouth, obviously caused by wearing false teeth on an illy fitting rubber plate. Some five weeks before, a little-pill fellow had bunglingly endeavored to extirpate it with a pair of scissors and table fork. It had grown very rapidly from the time of that mutilation, and was now as large as a walnut. Diagnosed it to be an epithelial cancer. Treatment.

℞. Chloride zinc,
Pulv. blood root, aa q. s.

Rub together in open air till it forms a stiff mass or paste. Put enough on cotton wool to cover the entire surface of the cancer, wedge more cotton wool between it and the upper lip, tell her to avoid allowing the saliva to come in contact with it as much as possible, and instruct her to remove it and have the paste wiped entirely out of the mouth when she has had it there long enough to become tired.

December 11th. Patient had left the paste in position about two hours, and on examination, I found a thin white eschar over the entire surface of tumor, and detached and loose in places Pocketed some paste under the loose parts of eschar, and applied more on the surface as before. I returned in about two hours and removed it myself.

I repeated this process for ten days, each day removing detached parts of the eschar, and applying fresh paste, both by pocketing it under loose parts of the eschar and on cotton wool. At the end of this time the wound presented that freedom from cancerous particles that one soon learns to recognize in using this paste. The applications were quite painful at times, but not dreaded, as would have been the knife.

Case 2. Nils A., fifty years of age, from Forest City, sixty miles west of this place, came to my office December 21st, 1874. Has had a little scabby sore on his under lip the last six months; lately it has grown very rapidly, and has become very painful. It now occupies the entire under lip, is red and purple on its surface, and is seamed and squamous near its centre, discharging an acrid fluid. Diagnosis, epithelial cancer. December 22d.—Apply the paste. December 23d.—As the paste has become dry and baked, I remove it and apply a fresh quantity. December 24th.—Remove the paste and apply bread and milk poultices. December 30th.—The eschar comes away this morning on removing the poultice. It is two and a half inches long, three-fourths of an inch wide, and five-eighths of an inch thick, at its greatest diameter. Some of the cancer remaining near the right angle of the mouth, I apply the following:—

| | | |
|---|---|---|
| ℞. | Arsenious acid | ʒ ij |
| | Mucilage gm. acacia, | ʒ j. |

Make into a stiff paste, apply it over surface of the cancer, press a fold of lint down over it, and when it becomes hardened cut away surplus lint with scissors. This is Dr. Marsden's "Cancer Paste," and should be used on not more than one square inch of surface at one time. I like it where the greater part of a cancer has been removed, and only a point or two of it is left behind in the wound, as this paste has more of a special election for the cancer than the zinc paste, and will not destroy so much of the surrounding healthy flesh, even when used in excess. January 1st.—Removed this paste by soaking it off with warm water, and have applied poultices ever since. A deep line of demarcation is now formed around the remaining part of the cancer, and I expect it to drop out in a day or two more.

